# Bony Healing of Unstable Thoracolumbar Burst Fractures in the Elderly Using Percutaneously Applied Titanium Mesh Cages and a Transpedicular Fixation System with Expandable Screws

**DOI:** 10.1371/journal.pone.0117122

**Published:** 2015-02-23

**Authors:** Anica Eschler, Stephan Albrecht Ender, Katharina Schiml, Thomas Mittlmeier, Georg Gradl

**Affiliations:** Dept. of Trauma, Hand and Reconstructive Surgery, University of Rostock, Medical Center, Schillingallee 35, D-18057, Rostock, Germany; Mathematical Institute, HUNGARY

## Abstract

**Introduction:**

There is a high incidence of vertebral burst fractures following low velocity trauma in the elderly. Treatment of unstable vertebral burst fractures using the same principles like in stable vertebral burst fractures may show less favourable results in terms of fracture reduction, maintenance of reduction and cement leakage. In order to address these shortcomings this study introduces cementless fixation of unstable vertebral burst fractures using internal fixators and expandable intravertebral titanium mesh cages in a one-stage procedure via minimum-invasive techniques.

**Material and Methods:**

A total of 16 consecutive patients (median age 76 years, range 58–94) with unstable thoracolumbar burst fractures and concomitant osteoporosis were treated by an internal fixator inserted via minimum invasive technique one level above and below the fractured vertebra. Fracture reduction was achieved and maintained by transpedicular placement of two titanium mesh cages into the fractured vertebral body during the same procedure. Intra- and postoperative safety of the procedure as well as analysis of reduction quality was analysed by 3D C-arm imaging or CT, respectively. Clinical and radiographic follow-up averaged 10.4 months (range 4.5–24.5).

**Results:**

Stabilization of the collapsed vertebral body was achieved in all 16 cases without any intraoperative complication. Surgical time averaged 102±6.6 minutes (71–194). The postoperative kyphotic angle (KA) and Cobb angle revealed significant improvements (KA 13.7° to 7.4°, p<0.001; Cobb 9.6° to 6.0°, p<0.002) with partial loss of reduction at final follow-up (KA 8.3°, Cobb 8.7°). VAS (Visual Analogue Scale) improved from 7.6 to 2.6 (p<0.001). Adjacent fractures were not observed. One minor (malposition of pedicle screw) complication was encountered.

**Conclusion:**

Cementless fixation of osteoporotic burst fractures revealed substantial pain relief, adequate maintenance of reduction and a low complication rate. Bony healing after unstable osteoporotic burst fractures is possible.

**Trial Registration:**

www.germanctr.de DRKS00005657

## Introduction

Vertebral compression fractures (VCF) affect 20% of people over the age of 70 with an increasing incidence [1–5]. The progressive character of osteoporotic spine deformation leads to a second fracture in 20% of patients with previous VCF within a 1-year period [6]. Besides pain with reduced mobility and quality of life those adjacent fractures lead to progressive kyphosis with a rise in morbidity and mortality rates due to decreased pulmonary function and abdominal constriction [7]. Most VCF occur after minor trauma rather than gradual accumulation of fatigue damage leading to an on-going process of creep [8,9]. A history of trauma has an impact on the severity of the fracture pattern. Most studies focus on MRI diagnosis in addition to plain x-rays. Recent studies on VCF in the elderly population using CT scans, however, clearly demonstrate a high number of unstable burst fractures in those patients that have not been involved in major trauma. Unstable fractures of the thoracolumbar junction are widely treated either by kypho/vertebroplasty or by a combination of both internal fixation and intravertebral cement application [10–14]. The latter option resembles to the treatment principles of the younger population. For younger patients the treatment goal of bony healing of the damaged vertebral body is mandatory. For the elderly, however, the chance for bony healing of compression or burst fractures has not been reported in the literature extensively [15]. Cement-based treatment algorithms play the major role in this age group regardless of the fracture severity. Fractures that involve the dorsal wall are at risk for cement-associated problems like leakage into the disc space or even into the epidural space besides physical cement-associated problems like exothermic reactions during the polymerization progress that might injure adjunct neuro-vascular structures [11,13,16,17]. Internal fixation devices turn to break out or displace in patients with reduced bone quality [18]. Having this in mind, current solutions in the treatment of osteoporotic burst fractures of the elderly comprise multi level dorsal fusions and combination with vertebro- or kyphoplasty [19,20]. Multilevel fixations, however, bear certain risks in the elderly like soft tissue complications, infection and pedicle screw cut-out. Pedicle screw displacement may be addressed by screw augmentation that in turn has certain cement-associated disadvantages [21,22]. In case of revision surgery, augmented screws may not be easily removed and cement leakage during insertion of screws still remains a problem. In order to address these afore mentioned specific problems we treated osteoporotic burst fractures by two-level level internal fixation employing expandable screws. This procedure was combined with two intracorporal expandable titanium mesh cages for fracture reduction and maintenance of reduction. It was hypothesised that this specific fixation technique i) leads to adequate restoration of vertebral geometry ii) shows the same ability of pain reduction as in solution applying bone cement and iii) shows no increased rate of implant failure in 2-level fixations with concomitant osteoporosis.

## Material and Methods

### Ethics Statement

The study was approved by the local ethical committee (University of Rostock, Germany) under the document no. A 2012–0004 and is in accordance with the declaration of Helsinki. Informed consent was written. The protocol for this trial and supporting TREND checklist are available as supporting information; see S1 TREND Checklist and S1 Protocol.

Sixteen vertebral bodies in 16 consecutive osteoporotic patients with unstable burst fractures at the thoracolumbar spine were treated operatively and observed prospectively (07/2012–12/2013). The VCF was defined as unstable in case of dorsal wall involvement. Fracture fixation combined vertebral body height reduction using two titanium mesh implants (OsseoFix) and adjacent transpedicular stabilisation using a posterior spinal fixation system and expandable, polyaxial screws (OsseoScrew) leading to a two-level fixation. All procedures were carried out in a percutaneous MIS (Minimally Invasive Surgery) technique without any cement application.

Mean age of the patients (7 male, 9 female) was 76.0 years (range 58–94 years). Ten patients suffered from more than three secondary diagnoses (Table 1). Clinically, all patients presented with acute back pain following a low-velocity trauma without any sensomotor deficits. Radiographic imaging including CT or MRI scans revealed AO type A3.1 incomplete burst fractures in all cases. Twelve lumbar (75%) and 4 thoracic vertebrae (25%) were affected. In 13 patients the diagnosis "osteoporosis" was known due to DXA measurements well before the actual fracture occurred, all of those received adequate medical treatment already (bisphosphonates). All patients were considered as patients at-risk and non-amenable to successful conservative treatment due to imminent vertebra subsidence with mobilisation. However, informed consent included detailed alternative treatment options including further conservative treatment as well as vertebro-/ kyphoplasty techniques and open approaches.

**Table 1:** Pain level rated by VAS during the study period (0 = no pain, 10 = maximum pain).

Patient No.	Age	Gender	Secondary diagnosis **	Fractured vertebra	VAS0 p.o.d.	VAS1 p.o.d.	VAS3 p.o.d	VASfinal follow-up
1	94	♂	c, p, e	T12	6.5	6.2	4.5	3.0
2	86	♀	c, n, e	L1	8.6	9.2	2.3	1.3
3	74	♀	g, e	L4	9.3	6.5	7.0	4.9
4	81	♀	c, n, e	L1	2.4	0	2.0	3.9
5	60	♂	c, p, g, e	L1	7.6	8.0	6.0	4.1
6	69	♀	c, e	L3	10.0	9.3	8.9	5.7
7	73	♀	c, g, e	L1	4.9	1.7	5.0	0
8	71	♀	c, r, n, e	L2	9.7	9.1	3.0	4.4
9	84	♂	c, e	L1	10.0	9.1	7.4	1.8
10	76	♂	c, e	L1	8.5	5.2	4.8	4.7
11	74	♀	c, h, e	L1	7.7	6.5	4.4	3.1
12	58	♂	n, g, e	T12	6.5	4.2	2.0	1.5
13	64	♂	-	L1	7.6	5.2	4.0	0.7
14	81	♂	c, e	T12	4.5	4.5	4.5	3.0
15	89	♀	c, e	T12	10.0	1.0	0	0
∑					7.8 *	**6.1**	**4.4**	**3.2**

* p<0.001 0 p.o.d.- 1 p.o.d., p<0.001 1 p.o.d.- 3 p.o.d., p<0.001 0 p.o.d.– 3 p.o.d, p<0.001 0 p.o.d. / 1 p.o.d.—final follow-up.

** Secondary diagnosis: c cardiovascular, p pulmonary, r renal, h haematological, n neurological, g gastrointestinal, e endocrine.

Inclusion criteria were incomplete burst fracture AO type A3.1 of the thoracolumbar spine, clinically persistent back pain, radiographic subsidence after mobilisation (proven by X-ray and CT scans), involvement of the dorsal wall (instable fractures), low velocity trauma, osteoporosis. Exclusion criteria were sensomotor deficits, non-amenability to MIS technique, relevant spinal canal stenosis, severe trauma, further injury’s than the vertebral fracture, missing radiological or clinical follow-up, dementia.

**Fig 1:**
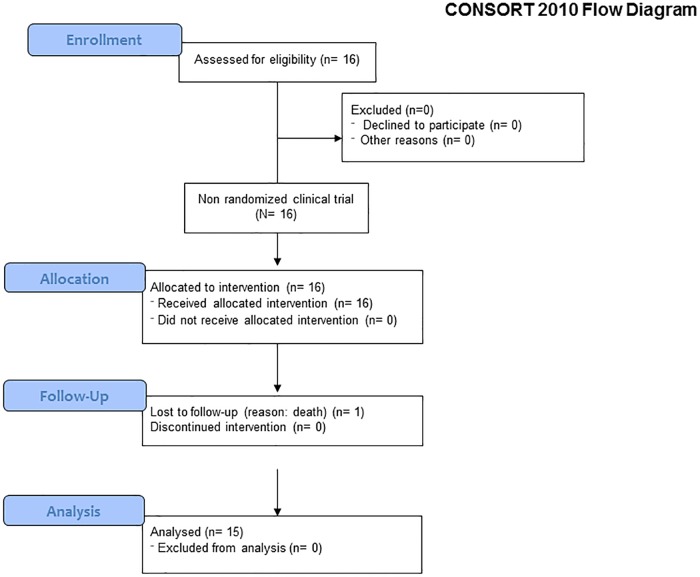
Consort 2010 flowchart.

Mean follow-up was 10.4 months (range 4.5–16.5). One patient had died due to reasons unrelated to the injury and intervention (cardiac failure) 1.2 years after surgery and was therefore lost for clinical follow-up (Fig. 1).

**Fig 2:**
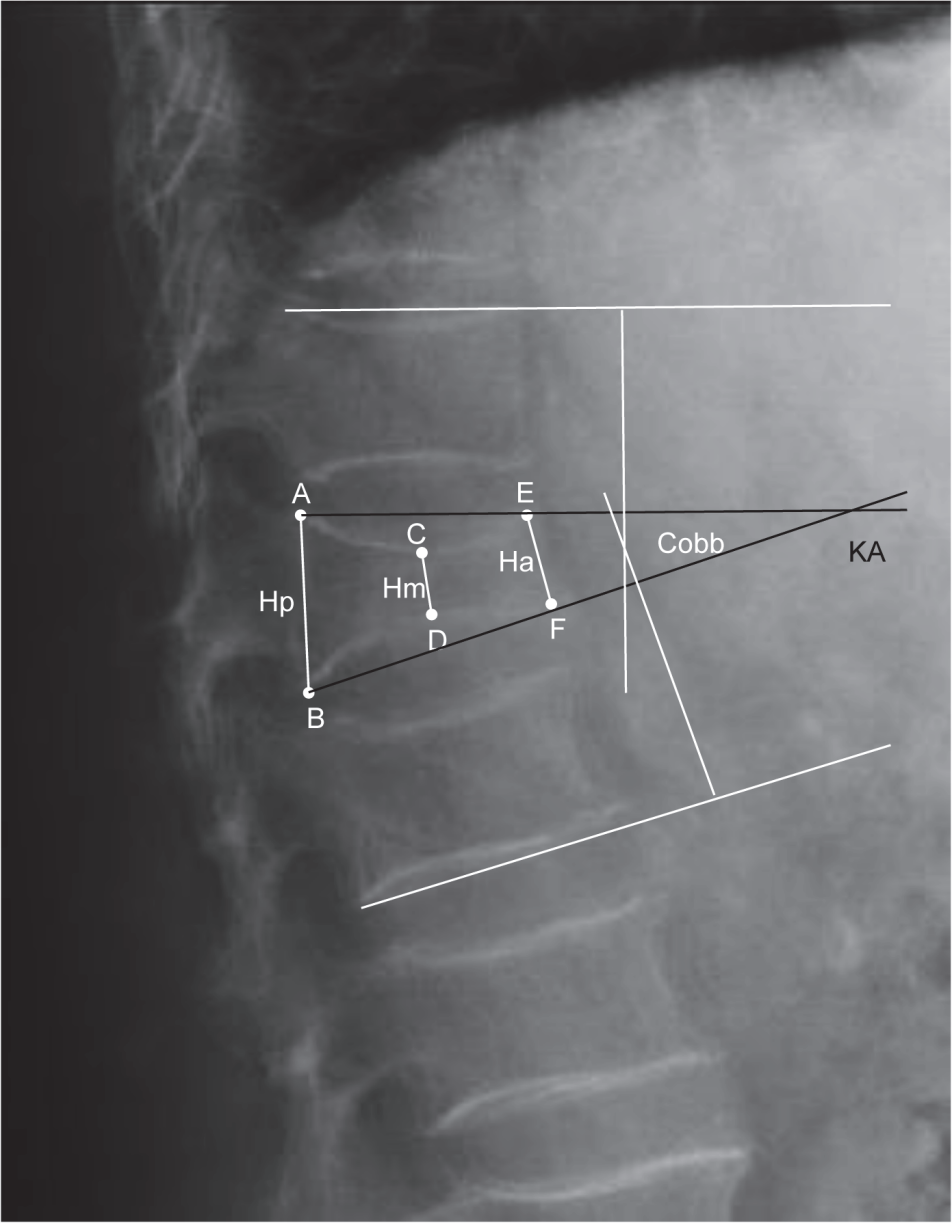
Radiographic measurement of vertebral body height in sagittal alignment using 6 defined points: A and B on the most dorsal-superior and—inferior endplate margins, E and F correspond to the most anterior-superior and—inferior margins, C and D are on the midpoint of a perpendicular line drawn from A to E and B to F on the superior and inferior vertebral endplates. Cobb- and vertebra body kyphotic angle.

Radiological follow-up included standing anterior-posterior and lateral radiographs at three different time points: preoperative, postoperative and for final follow-up (10.4 months postoperatively). In addition CT scans were available preoperatively; if necessary additionally 3D-fluoroscopy scans were performed intraoperative. All radiographs were analyzed for changes in sagittal alignment referring to the posterior (Hp), middle (Hm) and anterior body height (Ha) according to six defined points on the fractured vertebra (A-F, Fig. 2). The sagittal index (SI) as measurement of segmental kyphosis at the level of one spine segment was calculated from the posterior and anterior height. The vertebral body kyphotic angle (KA) was measured by means of the intersections connecting points AE and BF. The Cobb angle as measure for the sagittal alignment at the fracture level was calculated from the intersections of the tangents to the endplates of the corresponding vertebral bodies superior and inferior from the injured level. Preoperative MRI scans were abdicable with secure signs of recent fracture in CT scans and concomitant typical clinical symptoms. Bone healing tendency was observed on follow-up x-rays; in case of uncertain fracture union CT scans were performed for final follow-up (n = 2). Assumption of bony healing was defined by absence of signs of cage displacement or movement, surrounding dens bony structure and absent osteolysis of the cage surrounding tissue. All measurements were performed by an independent examiner with high expertise in spine surgery.

Clinical follow-up included pain rate evaluation referring to a Visual Analogue Scale (VAS) preoperatively, on the first and third postoperative days and at final follow-up. Furthermore, a standardized subjective and functional assessment of the spine including the Roland Morris Questionnaire (RMQ) [23], Oswestry Disability Index (ODI) [24], range of motion (ROM) measurement and testing of any sensory and motor deficits were performed at final follow-up.

### Surgical technique

In order to reduce and stabilize the collapsed vertebral body the patient was in a prone position and two limited surgical incisions (2 cm) were made lateral from the pedicle level after fluoroscopic identification of the fractured vertebra. With the help of a targeting needle two transpedicular K-Wires were placed into the vertebral body under fluoroscopic control in two planes, and the pathway into the anterior third of the vertebral body was drilled using a cannulated drill. The implant delivery system helped to insert the two implants (OsseoFix, Alphatec Spine, Carlsbad, California, US) which had been selected regarding their proper size according to preoperative planning from preoperative CT scan. To deploy the expandable cages in a controlled manner a mechanical actuation system was used. Bone cement was not applied in any case. Expandable pedicle screw implants (OsseoScrew, Alphatec Spine, Carlsbad, California, US) placement one level above and below the fractured body was performed in a percutaneous technique followed by bilateral connection of the ipsilateral pedicle screws with rods. No further compression or distraction was performed with the only reduction via patient positioning and the expandable cage. Transverse connectors were not employed, either.

Surgery was performed by three highly experienced spine surgeons; the follow-up was performed at the same institution (university trauma centre) by one single independent surgeon with advanced experiences in spine surgery.

Postoperatively, full weight-bearing and mobilization was allowed from the first postoperative day according to the patient’s pain threshold. For three patients that did not receive medical osteoporosis therapy prior to the vertebral fracture adequate therapy (calcium, vitamin D supplementation) was established and further diagnostics initiated.

### Statistical Analysis

Results were given as mean ± SEM (Standard Error of Mean) (range). After satisfying the assumption of normality (Kolmogorov-Smirnov), paired t-test analyses or Mann-Whitney U Test (non-normal distribution) were performed to analyse the differences in radiographic and functional parameters preoperative, postoperative and for final follow-up. According to the Bonferoni approach significance was defined at p < 0.025 or (0.05/2) for radiographic evaluation and p < 0.0167 or (0.05/3) for pain evaluation. Statistical testing was performed using IBM SPSS Statistics version 20.0 software (Armonk, New York, US). An a priori statistical power calculation was not conducted because the results presented are secondary to those of the chief purpose of the trial.

For confidentiality reasons this study’s registration according to WHO guidelines was not prior to patient enrollment. The authors confirm that all related trials for this intervention are registered.

## Results

**Fig 3:**
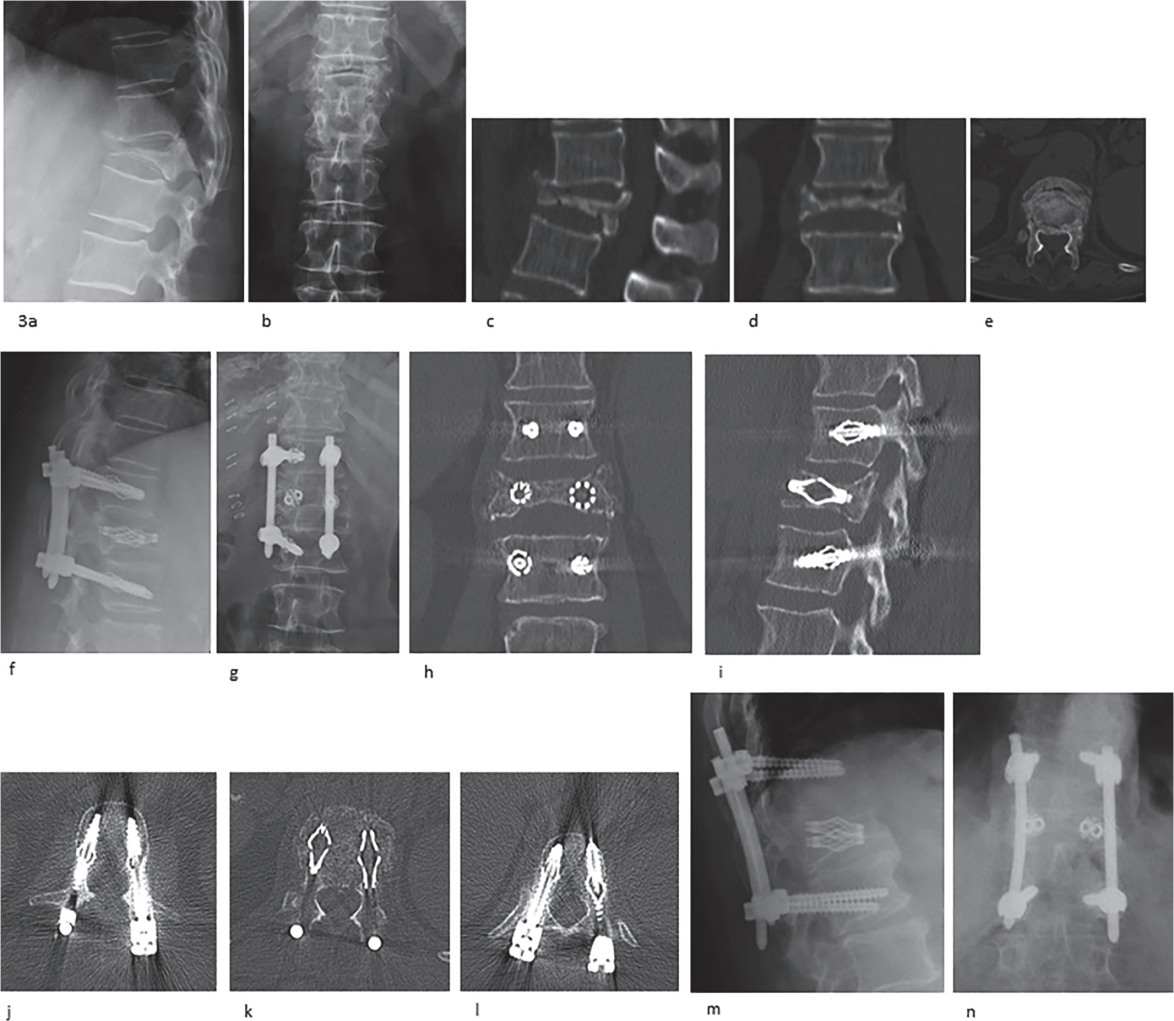
Radiographs pre- (a-e), post-surgery (f-l) and for final follow-up (m,n) showing a T1 burst fracture and operative treatment by an internal fixateur one level above and below the fractured vertebra body and reduction by transpedicular placement of two titanium mesh cages, all via minimum invasive technique.

Stabilization of the collapsed vertebral body was achieved in all 16 cases without any intraoperative complications (Fig. 3). Intraoperative 3D-Scans showed correct cage and screw positioning in all cases. Mean operation time was 102±6.6 minutes (71–194 minutes). Mean time of postoperative hospitalisation was 9.4±0.6 days (6–13 days); the preoperative hospitalisation period came up to 3.1±0.5 days (0–7 days).

### Radiological results

**Fig 4:**
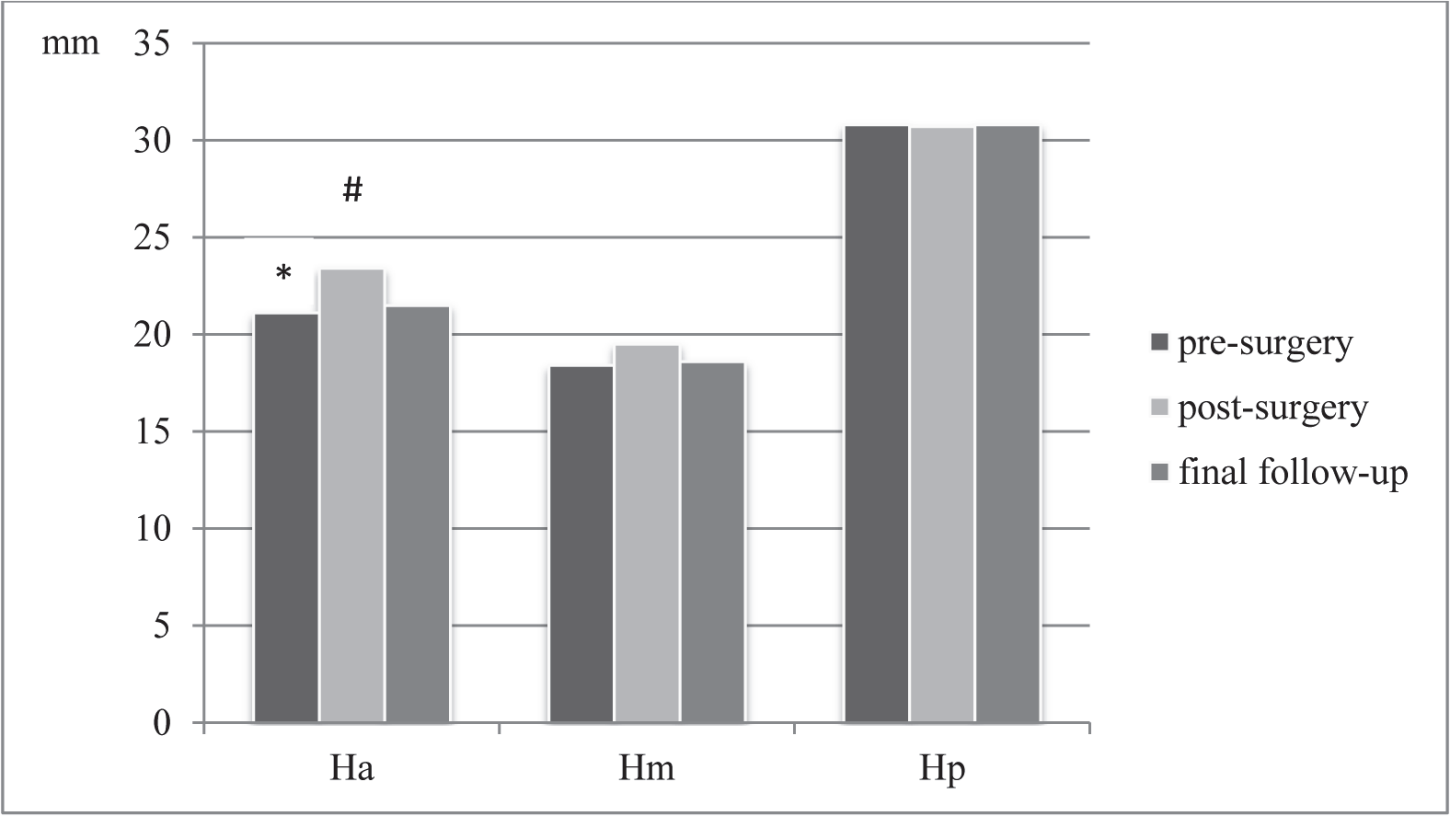
Radiographic evaluation for vertebral body reduction in sagittal alignment pre-/postoperatively and for final follow-up (Ha = anterior vertebral body height, Hm = middle vertebral body height, Hp = posterior vertebral body height). * pre-surgery vs. post-surgery p<0.033; # post-surgery vs. follow-up p<0.008.

Intraoperative vertebral body reduction was achieved in all 16 patients with respect to body angle and decrease of kyphotic angulation of the adjacent vertebra segments. Ha, as an indicator of vertebral body reduction, improved from 21.1±0.9 mm (16–28 mm) preoperatively to 23.4±1.0 mm (14–31 mm) postoperatively, but decreased to 21.5±1.1 (11–30 mm, p<0.008 (postoperative vs. follow-up)) at final follow-up (Fig. 4). Correspondingly, Hm improved from 18.4±0.7 mm (13–23 mm) to 19.5±1.0 (9–24 mm) and finally declined to 18.6±0.9 mm (8–23 mm). Hp did not show relevant changes (30.8±0.7 mm (25–37 mm) to 30.7±0.9 mm (14–31 mm) postoperatively to 30.8±0.9 mm (11–30 mm) at final follow-up. Consequently SI improved from 0.7±0.0 (0.5–1.0) to 0.8±0.0 (0.5–1.0) showing a tendency to less kyphosis of the vertebral body, however, re-kyphosis with a mean of 0.7±0.0 (0.4–1.0) SI was observed for final follow-up.

**Fig 5:**
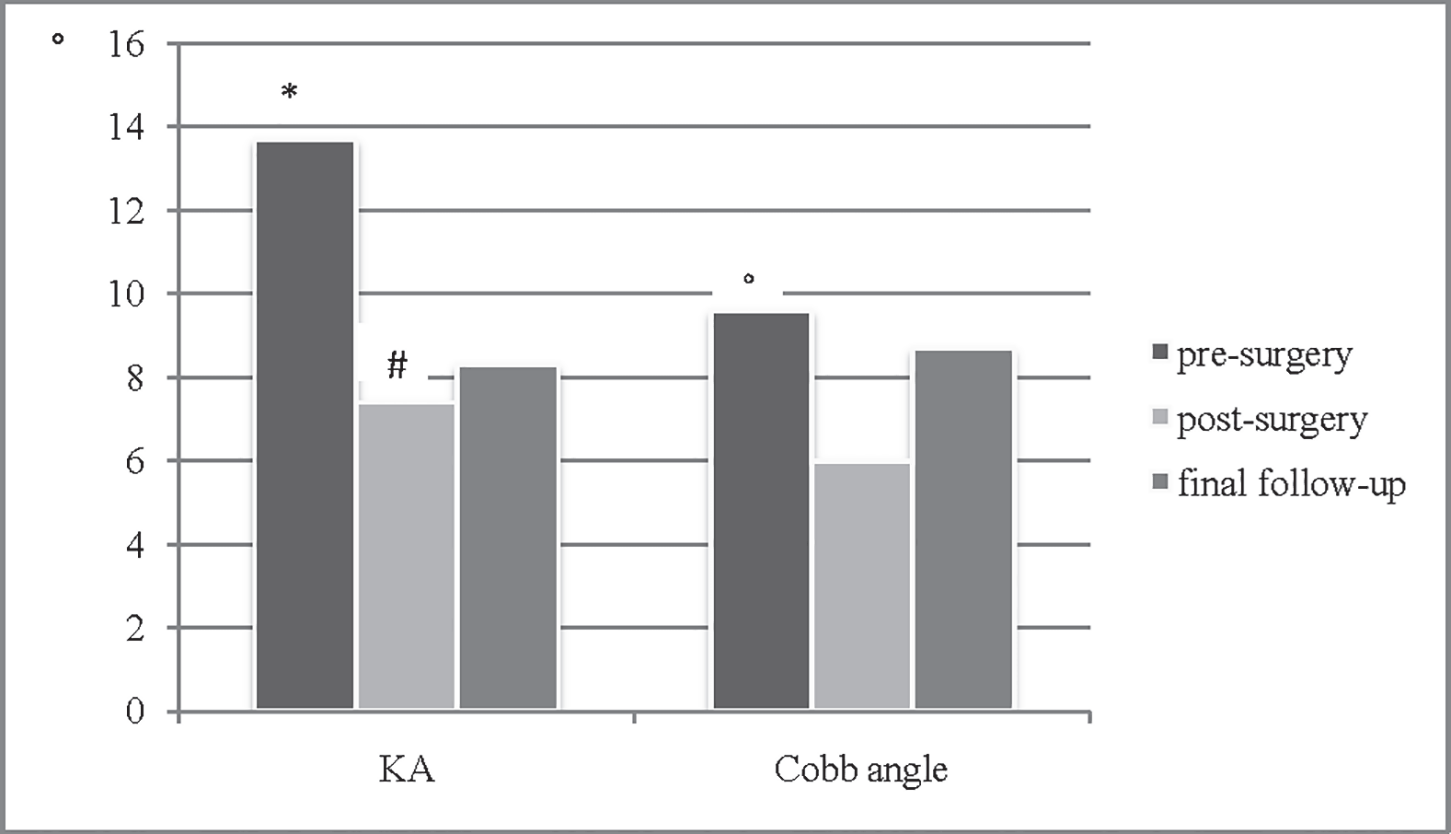
Radiographic evaluation for changes in vertebral body kyphotic angel (KA) and and Cobb angle alignment pre-/postoperatively and for final follow-up. * pre-surgery vs. post-surgery p<0.001, pre-surgery vs. follow-up p<0.001; # post-surgery vs. follow-up p<0.026;° pre-surgery vs. post-surgery p<0.002.

KA improved significantly from 13.7±1.2° (2–21°) to 7.4±0.8° (2–17°, p<0.001) revealing successful vertebral body reduction. Postoperative mobilisation led to a slight loss of reduction to 8.3±1.1° (2–18°, p<0.026 (postoperative vs. follow-up)) KA. Each vertebral body achieved a minimum reduction (KA) of 1.8°. Twelve patients (75%) achieved at least 5° improvement of whom 4 (25%) achieved more than 10° improvement in KA postoperatively (Fig. 5). In 7 cases (44%) no loss of reduction occurred. For the sagittal alignment of the below and above laying vertebral body segments was measured using the Cobb angle. The Cobb angle at the fracture level improved from 9.6±1.1° (2–28°) to 6.0±0.9° (1–22°, p<0.002) revealing less kyphosis. For final follow-up 8.7±1.7° (1–22°) angulation was measured.

Follow-up x-rays showed bony healing in 14 patients without further measurement methods, in two patients an additional CT-scan was necessary due to uncertain healing status showing callus development in progress 5 months postoperatively and complete bony healing after 6 months in one and 7.5 months in the other patient. During the whole study period we did not observe any adjacent vertebral fracture.

### Functional results

Pain as rated by the VAS scale accounted for 7.6±0.5 (2–10) preoperatively and improved significantly to 5.7±0.6 (0–9, p<0.001) directly after surgery. A further decrease to 4.1±0.5 (0–9) was measured on the third postoperative day, to 2.6±0.4 (0–6) for final follow-up. Detailed results are shown in Table 1.

Further clinical follow-up included the RMQ questionnaire as an indicator for pain and activities of daily living and resulted in a total of 7.6±1.5 points (1–19 points) which corresponds to only minor restriction, in dorsal extension at final follow-up. Results applying the ODI confirmed moderate disability with 27±0.1% (0–68%).

Mean range of motion in thoracolumbar flexion corresponded to 104±6.2° (20–120°) and to 17±3.0° (0–30°) in extension showing almost unaffected back motion. In analogy, rotation was measured rightwards with 21±2.0° (10–40°) and leftwards with 21±1.6° (15–30°). Lateral bending accounted for 24±2.4° (10–40°) rightwards and 21±2.0° (10–40°) leftwards. The minimum distance between fingertip and the floor was 11.8±3.8 cm (0–53 cm) in mean.

Postoperatively, all patients except one had full sensory and motor function (M5/5). One female patient experienced sensomotor deficits accounting for L3 left segmental nerve due to nerve root irritation resulting in muscle strength reduction of 3/5; although radiologically proven Ia pedicle screw positioning [25]. Within two months postoperatively the muscle strength improved to 4/5 (same result for follow-up). Another patient with T12 fracture and normal function for hospital discharge showed deficits in foot dorsal flexion at follow-up due to herniated disc development at a spinal level not related to the surgically addressed level.

### Complications

Postoperative CT scans revealed an intramedullary IIb malposition [25] of one pedicle screw which was asymptomatic, however, revision surgery for corrective replacement of the screw was performed. Functional rating for this patient came up to 10 points in RMQ, 42% in ODI and range of motion of 120° thoracolumbar flexion, 10° extension, 20°/20° rotation and 10°/20° lateral bending. Pain rating according to VAS for this patient accounted for 2.4 preoperatively, 0 postoperatively, 2.0 on the third postoperative day (after revision) and 3.9 for final follow-up.

## Discussion

The results of the present study indicate that bony healing of unstable osteoporotic vertebral fractures in the thoracolumbar spine is possible. A cementless stabilization technique using an expandable titanium mesh implant and a transpedicular fixation system with expandable screws allowed adequate primary reduction and stable fixation through a less invasive percutaneous instrumentation. Despite the fact that effective reduction of vertebral geometry could be achieved, reduction maintenance throughout the whole follow-up period was failed. MIS fixation technique led to a pronounced pain relief which was not adversely affected by loss of reduction.

The incidence of thoracolumbar burst fractures in the elderly is likely to be underestimated. The standard diagnostic parameters after low-velocity back trauma include x-ray and, if necessary, MRI evaluation which both do not securely show posterior wall involvement [8,9]. In case of a vertebral burst fracture at the thoracolumbar junction treatment mainly follows the rules of stable VCF treatment or a combination of vertebroplasty and internal fixation [17,19,20,22]. Under these circumstances only limited reduction of the vertebral body collapse is possible leading to limited improvement of sagittal spine alignment [26,27]. Kyphoplasty, is able to better restore vertebral height by means of a balloon but still fails primary reduction in KA angle radiologically in 34% [28], followed by a secondary loss of height in 18–63% [29,30]. Conservative therapy, which is in spite of instability criteria performed in some parts of the world, show equal results, however come along with the risk of development of neurological deterioration [31]. As shown in this study the combination of intervertebral mesh stent and dorsal spinal fixation system for burst fractures lead to an improved initial reduction in all cases of 6.3° KA angle (46%). In 9 cases (56%) a slight secondary loss of reduction of 0.9° (7%) was observed for follow-up. The Cobb angle improved considerably from 9.6° to 6.0°, the loss of reduction was limited to 8.7°.

Clinical outcome variables showed an average reduction in pain intensity based on the VAS, from 7.6 to 2.6 for final follow-up thus presenting comparable results to vertebroplasty-and kyphoplasty procedures (8.7–7.3 to 5.4–2.2) [19,32–34]. Among other parameters this may indicate that the additional MIS dorsal fixator did not adversely affect the postoperative pain relief. In line with this, ODI functional results improved in an analogous manner to vertebroplasty-and kyphoplasty. A metaanalysis of 69 clinical trials showed comparable results for pain relief and function for both, vertebro- and kyphoplasty [11,35–40]. In only 13 patients osteoporosis was diagnosed prior to the vertebral fracture, three patients did not receive medical osteoporosis therapy before. In those cases establishment of adequate therapy (calcium, vitamin D supplementation) and initiation of further diagnostics is essential for satisfying long term clinical results.

The general finding in this study of bony healing in osteoporotic burst fractures may not be as unusual as expected, although it does not play a major role in current clinical treatment algorithms since kyphoplasty and vertebroplasty are both based on PMMA cement application for fracture stabilisation which in certain degrees disables bony healing capacity. Bony consolidation around the cement block may occur, however, it might be more likely that the cement had adverse effects on the bony consolidation process [41]. Xu et al. [42] did not show in an experimental setting of osteoporotic fracture healing (conservative) an increased non-unions rate, nevertheless quantity and quality of callus mineralization was decreased. Similar results were seen in a femoral fracture model in osteoporotic rats displaying a 40% reduction in fracture callus cross-sectional area and a 23% reduction in bone mineral density, but still uneventful healing [43].

In addition, cement application changes the biomechanical properties of the vertebral body considerably which may in turn promote other complication like adjacent fracture development [44–50]. Adjacent vertebral fractures are the most common complications with up to 8% after vertebroplasty and 8 to 26% after kyphoplasty reported in literature [16,36,51–55]. We did not observe any adjacent fracture during the admittedly short follow-up in our study collective.

Another severe problem associated with bone cement might be cement leakage into the epidural space causing neurologic symptoms with significantly increased rates in fractures affecting the dorsal vertebral wall [13,36,51]. Uncontrolled cement leakage into the venous system due to the high pressure forces used while application may lead to pulmonary embolism. 4 to 13% leakage rate for kyphoplasty and 20 to 70% for vertebroplasty were described in literature [38,51,56,57]. With cementless application of the expandable titanium mesh cage and the dorsal spinal fixation system we did not see any of these complications leading to a low complication rate when compared to standard methods [11,13,16,17]. Instead, we saw one case with an asymptomatic pedicle screw malposition leading to revision surgery.

The weaknesses of this study focus on the radiographic evaluation with its putative measurement error. Anyhow, the method was based on the current literature and seems to be a general problem due to the lack of superior methods [26,58].

## Conclusion

Preliminary results indicate the high likelihood of bony healing in osteoporotic fractures of the thoracolumbar spine when using intravertebral titanium cages without cement application. The addition of a percutaneous internal fixator employing expandable pedicle screws allowed for a two level fixation with reduced risk of screw cutout while cement associated complications cease to exist with this technique. Significant pain relief and good functional results indicated that minimum invasive application of an additional internal fixator did not deteriorate functional outcome. However, maintenance of reduction was not successful in all cases leading to similar results compared to standard kypho/vertebroplasty with this matter.

## Supporting Information

S1 TREND Checklist TREND checklist. (PDF)

S1 Protocol Study protocol (English). (DOC)

S2 Protocol Study protocol (original language, german). (DOC)
