# Correction: Bony Healing of Unstable Thoracolumbar Burst Fractures in the Elderly Using Percutaneously Applied Titanium Mesh Cages and a Transpedicular Fixation System with Expandable Screws

**DOI:** 10.1371/journal.pone.0124591

**Published:** 2015-04-07

**Authors:** Anica Eschler, Stephan Albrecht Ender, Katharina Schiml, Thomas Mittlmeier, Georg Gradl

The affiliation for the second author is incorrect. Stephan Albrecht Ender is affiliated with: Department of Orthopaedics and Orthopaedic Surgery, University Medicine Greifswald, Ferdinand-Sauerbruch Straße, 17475 Greifswald, Germany.
