# Supplementary material for: Bony Healing of Unstable Thoracolumbar Burst Fractures in the Elderly Using Percutaneously Applied Titanium Mesh Cages and a Transpedicular Fixation System with Expandable Screws
Source: PLoS One. 2015 Feb 23;10(2):e0117122. doi: 10.1371/journal.pone.0117122 (PMC4338244; doi:10.1371/journal.pone.0117122)
Supplement: S1 Protocol — (DOC) [file pone.0117122.s002.doc]

Study protocoll to

**“Bony Healing of Unstable Thoracolumbar Burst Fractures in the Elderly Using Percutaneously Applied Titanium Mesh Cages and a Transpedicular Fixation System with Expandable Screws”**

– translated to english

## Methods

Patients with burst or compression fractures of the thoracolumbar spine (AO A3.1.) and specific indication for operative therapy will be treated operatively using the “Osseofix” implant in standalone technique or a combination of “Osseofix” and an internal fixator (“Ilico”). Preoperative evaluation includes the Oswestry Disability Questionaire, Roland Morris Disability Questionnaire and pain rate evaluation using a visual analogue scale. Additionally, radiographs in two plans and a CT scan of the injured spine segments will be performed. Postoperative evaluation includes the patient’s pain rate (visual analogue scale) radiographic evaluation using radiographs in two plans and a CT scan. A detailed functional evaluation is performed six and twelve months after surgery including inspection, palpation, testing of range of motion, muscle function and sensory function and again the patients have to answer the Oswestry Disability Questionaire and Roland Morris Disability Questionnaire. For this point in time, again, radiographs in two plans for evaluation of complications as adjacent vertebral body fractures will be performed. Since financial limitations in the ambulant sector do not always allow for control radiographs they will be performed at the investigators clinic if not available.

The investigation will include 20 patients.

## Analysis

Statistical analysis will be performed using a customary notebook and in collaboration with a statistician. Data will be saved using the program Excel and statistical evaluation performed using the program SPSS.
