# Supplementary material for: Bony Healing of Unstable Thoracolumbar Burst Fractures in the Elderly Using Percutaneously Applied Titanium Mesh Cages and a Transpedicular Fixation System with Expandable Screws
Source: PLoS One. 2015 Feb 23;10(2):e0117122. doi: 10.1371/journal.pone.0117122 (PMC4338244; doi:10.1371/journal.pone.0117122)
Supplement: S2 Protocol — (DOC) [file pone.0117122.s003.doc]

Study protocoll to

**“Bony Healing of Unstable Thoracolumbar Burst Fractures in the Elderly Using Percutaneously Applied Titanium Mesh Cages and a Transpedicular Fixation System with Expandable Screws”**

– original version, german

## Methodik

Patienten mit thorakolumbalen Wirbelkörperberstungs- und Kompressionsfrakturen werden bei gegebener Indikation mit „Osseofix Standalone“ oder Kombination aus „Osseofix“ und internem Fixateur („Ilico“) behandelt (AO A3.1). Präoperativ untersucht, erhalten die Patienten u.g. Fragebogen Oswestry- Disability- Index und Roland Morris Disability Questionnaire und machen Angaben zum Schmerzniveau anhand einer VAS (vis. Analogskala). Zudem werden Röntgenaufnahmen in zwei Ebenen und ein CT der betroffenen Wirbelhöhe durchgeführt. Direkt postoperativ sollen die Patienten erneut Angaben anhand der VAS machen und erhalten erneut Röntgenkontrollen in zwei Ebenen sowie ein CT. Sechs und 12 Monate postoperativ werden die Patienten schriftlich einbestellt und einer körperlichen Untersuchung unterzogen. Die Untersuchung umfasst Inspektion, Palpation der Wirbelsäule, Messung der Bewegungsumfänge, Muskelfunktionstest, Sensibilitätsprüfung und Ermittlung des Oswestry- Disability- Index und Roland Morris Disability Questionnaire – Scores. Zu diesem Zeitpunkt erfolgen erneut Röntgenaufnahmen des operierten Wirbelsäulenbereichs in zwei Ebenen um mögliche Anschlußfrakturen auszuschließen. Da die Einsparungen im Gesundheitswesen das dem weiterbehandelnden niedergelassenem Kollegen nicht immer ermöglichen werden die Röntgenaufnahmen in der untersuchenden Klinik gemacht, es sei denn, es liegen auswärtig durchgeführte Aufnahmen vor.

Es werden mindestens 20 Patienten untersucht.

## Auswertung

Die Auswertung der Daten erfolgt statistisch auf einem handelsüblichen Laptop. Dafür ist die Mitarbeit einer Statistikerin/ eines Statistikers vorgesehen. Die Daten werden in einer Excel-Datei geführt und statistisch mit dem Programm SPSS (Version 15.0) ausgewertet.
